# Editorial: The Individual Microbe: Single-Cell Analysis and Agent-Based Modelling

**DOI:** 10.3389/fmicb.2018.02825

**Published:** 2018-11-21

**Authors:** Johan H. J. Leveau, Ferdi L. Hellweger, Jan-Ulrich Kreft, Clara Prats, Weiwen Zhang

**Affiliations:** ^1^Department of Plant Pathology, University of California, Davis, Davis, CA, United States; ^2^Water Quality Engineering, Technical University of Berlin, Berlin, Germany; ^3^Centre for Computational Biology & Institute of Microbiology and Infection & School of Biosciences, University of Birmingham, Birmingham, United Kingdom; ^4^Department of Physics, School of Agricultural Engineering of Barcelona, Universitat Politècnica de Catalunya–BarcelonaTech, Castelldefels, Spain; ^5^Laboratory of Synthetic Microbiology, Key Laboratory of Systems Bioengineering (Ministry of Education), School of Chemical Engineering and Technology, Tianjin University, Tianjin, China

**Keywords:** individual microbe, single-cell analysis, individual-based ecology, agent-based modeling, research topic

The field of microbial individual-based ecology, or μIBE (Kreft et al., 2013), is grounded in the notion that to truly understand the interactions of microorganisms with their biotic and abiotic environment, one cannot ignore the scales at which such interactions occur. The collection and interpretation of data along these scales (from very small spatial dimensions to very large population sizes) remains a major challenge. Embracing the idea that “progress in science depends on new techniques, new discoveries, and new ideas, probably in that order” (Brenner, 2002), we introduce here a collection of 14 articles authored by 65 leading experts on the topic of “The Individual Microbe” (https://www.frontiersin.org/research-topics/5193). We frame these articles in a narrative that explores the progress made on techniques that extract and process information from individual microbes (IMs) and their environment, how that information allows the discovery and prediction of novel single-cell behaviors, and how those discoveries might generate new ideas about the outcomes and impacts of microscopic activity at macroscopic levels.

## New techniques

Techniques of importance to μIBE fall into one of two categories that we refer to here as “wet” and “dry.” Both types allow the observation and interrogation of IMs and their surroundings, but wet techniques do so of “real” IMs (i.e., bacteria, yeast, fungi, protists, etcetera) in a laboratory or field setting, while dry techniques involve virtual IMs (or “agents”) in computer-simulated environments (Kreft et al.). For wet approaches, much of the recently reported progress relates to increased compatibility with other methods that interrogate the same IM for multiple attributes or experiences, or at increasingly finer spatial or temporal resolution and/or with higher throughput. Some examples are highlighted here. While flow cytometry is a very useful high-throughput interrogation technique, it is incompatible with single-cell interrogation methods that are not fluorochromogenic. Guo et al. describe a variation of flow cytometry called “mass cytometry” where fluorochromes are replaced with heavy metal ions and which allowed the researchers to establish a direct link between the uptake of (antibacterial) silver and cell viability at the single-cell level. Harrison and Berry compare applications of vibrational microspectroscopy (a combination of spectroscopy and microscopy) for chemical imaging of microbial cells and their surroundings at high resolution and high throughput. The compatibility of Raman and Fourier-transform infrared imaging with other methods such as fluorescent *in situ* hybridization, stable-isotope probing, secondary ion mass spectrometry, and X-ray computed tomography makes it possible to extract local environmental context for the behavior or location of IMs. Such context can help with the interpretation of heterogeneity among a population of IMs as inherent to the IMs or in response to environmental heterogeneity (see below). Chen et al. review tools for DNA- and RNA-based single-cell analysis, which range from low and medium resolution (DAPI staining of chromosomal DNA to count IMs and transcriptional fusions to a reporter such as *gfp* to monitor expression of a single gene of interest, respectively) to high resolution (whole-genome or whole-transcriptome sequencing of single cells). Tools like the latter are still relatively new and face formidable technical challenges. Yet, they offer great hope not only for assessing single-cell variation in DNA and RNA content, but also for unlocking nucleotide-based information from hard-to-culture microorganisms.

As for dry μIBE techniques (i.e., agent-based modeling approaches), key advances often allow handling larger numbers of IMs per time unit, more attributes per IM, or including IMs representing multiple taxa or guilds. Such advances may be achieved by increasing computing power (for example Wilmoth et al.) or by structuring models more efficiently for faster (re-)calculations of location, perception, and response of IMs. Sometimes, the bottleneck for model progress is the lack of experimental data. For example, Garcia et al. measured cell volumes of exponentially growing bacterial cells using flow cytometry, and were able to derive parameters for a stochastic model of cell elongation and division to predict bacterial population growth. Similarly, Ginovart et al. used digital image analysis to determine yeast growth under different oxygen concentrations and to parameterize individual behavior into an agent-based model for the interpretation of population-level measurements. Often, agent-based models are improved by introducing additional layers of complexity, for example by modeling the behavior and interactions of IMs as a function of the behavior and interactions of cellular components within each IM (Kreft et al.).

## New discoveries

One major discovery in recent years is the existence and extent of heterogeneity among IMs. González-Cabaleiro et al. explore the magnitudes, sources and consequences of such heterogeneity, in particular as it pertains to bioprocess industries and design, where such heterogeneity has macroscopic consequences. Zhao et al. demonstrated this for IMs in clonal populations of the beer-spoilage bacterium *Lactobacillus brevis*. While all cells in populations of hop-sensitive strains were classified as dead after exposure to (antimicrobial) hops, a small fraction of cells in populations of hop-tolerant strains was alive and responsible for the tolerant phenotype. This type of heterogeneity is referred to as intrinsic, i.e., inherent to the IM, as opposed to extrinsic heterogeneity. An example of the latter is Nieß et al. who showed that long mixing times in large-scale bioreactors cause spatial variation in nutrient availability, which in turn triggered heterogeneity in the starvation response among microbes in the reactor. Intrinsic and extrinsic heterogeneity are often coupled. For example, Tack et al. combined an individual-based modeling framework with a metabolic simulation of the bacterium *Escherichia coli* to show that local differences in bacterial activity (i.e., oxygen consumption) lead to local differences in responses of those bacteria (i.e., secretion of weak acid products) generating local differences in environmental conditions (i.e., pH). Oftentimes, heterogeneity among IMs in their natural environment is observed, but its intrinsic or extrinsic nature is not well-understood. Ben Rejeb et al. used GFP-based bioreporters to show significant variation in gene expression among individual cells of a *Bacillus thuringiensis* population during infection of the host insect species *Galleria mellonella*. But is this heterogeneity due to the variation in the IM's experience of different microenvironments inside the insect (is it extrinsic heterogeneity?), or does it represent intrinsic heterogeneity, where variation in gene expression is hardwired into the *B. thuringiensis* way of life, representing what is known as programmed heterogeneity (Kreft et al.)?

Especially exciting (and challenging) are new discoveries that follow from observations of IMs and that defy or generate expectations. An example is El-Kirat-Chatel et al. who used atomic force microscopy to quantify the surface adhesion of bacteria at the single cell level. Surprisingly, their measurements did not correlate well with the adhesion forces measured at the population level. Do such unexpected observations expose fundamental flaws in our ability to scale microbial behaviors? How about agent-based models that accurately describe and validate one type of lab-observed IM behavior, but then also predict another type of behavior, one that has never been seen before, but that if experimentally confirmed would challenge existing theories and/or generate new ideas about the biology and ecology of IMs?

## New ideas

When asked about the field of biology, the philosopher Rudolf Carnap offered a definition (Carnap, 1938) that referred to “the behavior of individual organisms and groups of organisms within their environment.” This distinction between individual and group is, in a nutshell, the big idea behind μIBE. As a discipline, microbiology has long relied on population-based measurements, with little regard for the fact that life at the microscale is different from life as humans experience it (Dusenbery, 2011). Different rules apply at that scale, many of which are not intuitive and involve laws of small forces, large numbers, and unexpected probabilities. In reference to the latter, Jayathilake et al. used an agent-based model of 2-dimensional bacterial biofilm formation to test outcomes of single-cell variation in the ability to produce extracellular polymeric substances and to engage in quorum sensing. The study showed that chance played a key role in the outcome of the simulations, as the structure of the biofilm was partly determined by the initial random colonization of bacteria on the surface. For experimentalists, chance is difficult to accept as a driving force behind outcomes. Moreover, chance events at the micrometer scale are hard to control for when most experimental techniques do not allow high-resolution manipulation of single cells or their immediate environment. This frustration is in part what underlies many recent improvements in wet μIBE technology that deal with the construction of experimental arenas where such micro-manipulation is possible (for example, microfluidic setups like the one used by Wilmoth et al.).

And so, Brenner's postulate (Brenner, 2002) certainly rings true for the field of μIBE, where progress crucially depends on new techniques, both wet and dry, not only to allow new discoveries about the existence and extent of heterogeneity between IMs, but also in pursuit of testing the idea that such heterogeneity matters.

## Author contributions

FH, J-UK, CP, and WZ wrote summaries of the papers published on this Research Topic. JL wrote the first draft of the manuscript, which was read and revised by the other authors. All authors approved the submitted version.

### Conflict of interest statement

The authors declare that the research was conducted in the absence of any commercial or financial relationships that could be construed as a potential conflict of interest.

## References

[B1] Brenner S. (2002). Detective Rummage investigates. Scientist 16:15.

[B2] Carnap R. (1938). Logical foundations of the unity of science, in International Encyclopedia of Unified Science, Vol. I, Part 1, eds Neurath O. Carnap R. Morris C. W. (Chicago, IL: University of Chicago Press), 42–62.

[B3] Dusenbery D. B. (2011). Living at Micro Scale: the Unexpected Physics of Being Small. Harvard, IL: Harvard University Press.

[B4] Kreft J.-U. Plugge C. M. Grimm V. Prats C. Leveau J. H. J. Banitz T.. (2013). Mighty small: observing and modeling individual microbes becomes big science. Proc. Natl. Acad. Sci. U.S.A. 110, 18027–18028. 10.1073/pnas.1317472110 24194530 PMC3831448

